# Development of a Rapid and Simple Method to Remove Polyphenols from Plant Extracts

**DOI:** 10.1155/2017/7230145

**Published:** 2017-10-23

**Authors:** Imali Ranatunge, Subshini Adikary, Piumi Dasanayake, Chamira Dilanka Fernando, Preethi Soysa

**Affiliations:** Department of Biochemistry and Molecular Biology, Faculty of Medicine, University of Colombo, Colombo, Sri Lanka

## Abstract

Polyphenols are secondary metabolites of plants, which are responsible for prevention of many diseases. Polyvinylpolypyrrolidone (PVPP) has a high affinity towards polyphenols. This method involves the use of PVPP column to remove polyphenols under centrifugal force. Standards of gallic acid, epigallocatechin gallate, vanillin, and tea extracts* (Camellia sinensis)* were used in this study. PVPP powder was packed in a syringe with different quantities. The test samples were layered over the PVPP column and subjected to centrifugation. Supernatant was tested for the total phenol content. The presence of phenolic compounds and caffeine was screened by HPLC and measuring the absorbance at 280. The antioxidant capacity of standards and tea extracts was compared with the polyphenol removed fractions using DPPH scavenging assay. No polyphenols were found in polyphenolic standards or tea extracts after PVPP treatment. The method described in the present study to remove polyphenols is simple, inexpensive, rapid, and efficient and can be employed to investigate the contribution of polyphenols present in natural products to their biological activity.

## 1. Introduction

Polyphenols are abundant in natural food sources. The ability to scavenge reactive oxygen species associates with beneficial effects of polyphenols on human health [1]. Recent studies on natural food and herbal medicine show that there is an increasing scientific interest towards the role of polyphenols in health.

Polyvinylpolypyrrolidone (PVPP) is a water insoluble polymer which adsorbs polyphenols via hydrogen bonding and other weak forces [2]. PVPP has a high capacity to bind polyphenolic compounds such as catechin, epicatechin, and quercetin [3, 4]. Further PVPP has been used to remove polyphenols from beverages to improve the quality of transgenic* Lemna minor* extracts expressing mAb [5]. Recent study carried out by Hernández-Bolioa et al. [6] has compared four different methods to remove polyphenols from* L. latisiliquum* to study the effect on the biological activity of PVPP adsorption, liquid–liquid partition with aqueous NaCl, polyamide-SPE extraction, and gel permeation chromatography using Sephadex LH-20. Similar method to current study has been developed to quantify caffeine from tea using a PVPP pretreatment cartridge. This study employs a SPE vacuum manifold which is an expensive attachment [7].

Several studies have shown that there is an association between antioxidant activity and polyphenol content [8–10]. The removal of polyphenols prior to the assay will confirm whether the biological activity is attributed to polyphenols, which is lacking in most studies reported. A PVPP column was used in the present study to adsorb polyphenols and the remaining solution passing through the column was collected to study the contribution of polyphenols to the bioactivity of natural products. The technique described in the present protocol involves a further improvement and optimization of the method developed in our laboratory for removal of polyphenols from natural products using black tea extracts* (Camellia sinensis)* as the model system [11, 12]. The present study employs a single step to remove polyphenols efficiently and quickly.

## 2. Materials and Methods

### 2.1. Chemicals and Equipment

polyvinylpolypyrrolidone (PVPP), Folin-Ciocalteu reagent, gallic acid (GA), *β*-hydroxyethyl theophylline, caffeine, epicatechin (EC), (−)-epigallocatechin gallate (EGCG), and 1, 1- diphenyl-2-picrylhydrazyl (DPPH) purchased from Sigma Chemicals, USA. Acetonitrile (HPLC grade) was purchased from BDH chemicals. HPLC was performed with Shimadzu LC 10AS solvent delivery system equipped with UV/VIS detector Shimadzu SPD 10A and an integrator Shimadzu C-R8A (Shimadzu Corporation, Japan). Betasil phenyl HPLC column (2.1 × 150 mm) (Thermo scientific) was used to separate catechins and caffeine. Centrifugation was performed using a BioFuge-Pico D-37520 centrifuge (Heraeus Instruments, Germany).

### 2.2. Preparation of Tea Extracts

Commercially available black tea and freeze dried fresh tea flush (bud and adjoining two leaves) were used in this study. Black tea leaves (10 g) were boiled with 400 mL of deionized water. Extract was filtered through a cotton wool plug to a volumetric flask and made up to 500 mL. Fresh tea flush collected from Akuranekande Tea Estate Ehaliyagoda, Sri Lanka. Dried tea flush (1 g) was sonicated in deionized water (8 mL) for 2 hours and centrifuged at 9000 rpm for 20 minutes. The supernatant was filtered through a Whatman number 1 filter paper. Freshly prepared tea extracts were used for HPLC analysis and to determine the total phenolic content.

### 2.3. Optimization of PVPP for Removal of Polyphenols from Tea Extracts

**Figure 1:**
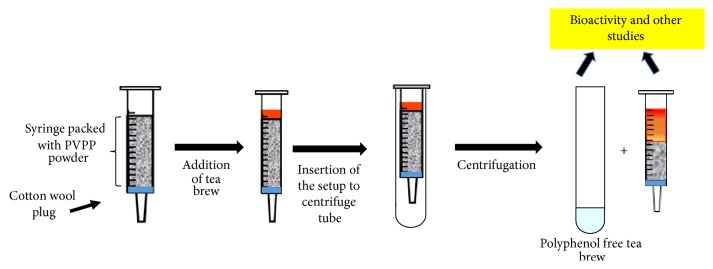
Separation of polyphenols from natural products using a PVPP packed column.

A cotton wool plug was placed at the bottom of the syringe (5 cc) after removing the plunger and the needle. Different quantities of PVPP were used to optimize the extraction procedure at room temperature. PVPP powder (0.9 g, 0.7 g, and 0.5 g) was added on the top of the cotton wool plug and the syringe was placed on a centrifuge tube. Tea extracts (2 mg/mL; 3 mL) were layered over the PVPP column as depicted in Figure 1. The column loaded with tea extract was centrifuged at 2000*g* for 10 minutes and the supernatant was collected (fraction). The first fraction was discarded (fraction 1). This procedure was repeated with 1 mL of tea extract for 6 times and each fraction was collected into separate tubes. Crude tea extract and each fraction collected were analyzed for total polyphenols and the presence of tea constituents (caffeine and catechins). The experiment was carried out in replicates (*n* = 6). The same procedure was followed for standard solutions of GA, EGCG, and vanillin (*n* = 3).

### 2.4. Determination of Total Polyphenols (TPC)

TPC of tea extracts and polyphenol free fractions collected in each cycle were quantified by Folin-Ciocalteu (F-C) method [13]. Absorbance for the standards of gallic acid, epigallocatechin gallate (EGCG), and vanillin (100 *μ*g/mL) was also read at 760 nm after performing F-C assay before and after PVPP treated (PVPPT) samples.

### 2.5. Determination of UV Absorbance

The absorbance of standards (100 *μ*g/mL) of gallic acid, epigallocatechin gallate (EGCG), and vanillin present in the sample before and after the removal of polyphenols was measured at 280 nm using a spectrophotometer with deionized water as the blank (*n* = 3). Samples of fresh tea flush (1 mg/mL) were also carried out simultaneously.

### 2.6. Analysis of Gallic Acid, Caffeine, EC, and EGCG in the Fresh Tea Flush before and after Treatment with PVPP

Reverse phase high performance liquid chromatography (RP-HPLC) was carried out to detect gallic acid, caffeine, EC, and EGCG standards (each at 8 *μ*g/mL) in the tea extract and PVPPT tea extracts as previously described [14]. Tea constituents were separated using 8% acetonitrile in 1% glacial acetic acid as the mobile phase at a flow rate of 0.5 mL/min on a phenyl column. *β*–Hydroxyethyl theophylline (10 *μ*g/mL) was used as the internal standard. Tea extracts and PVPPT tea extracts and standards (100 *μ*L) were mixed with *β*–hydroxyethyl theophylline (100 *μ*L) followed by centrifugation and the supernatant (25 *μ*L) was injected onto the column. A standard composed of gallic acid, caffeine, EC, and EGCG was simultaneously run with test samples. Peaks were identified by their retention time and spiking the authentic sample. The peaks were detected at 280 nm.

### 2.7. Analysis of Standards of Gallic Acid, Vanillin, and EGCG before and after Treatment with PVPP

Presence of gallic acid, vanillin, and EGCG (100 *μ*g/mL) following PVPP treatment was analyzed and compared with their standards (100 *μ*g/mL) using HPLC as described above in the absence of the internal standard.

### 2.8. Determination of Antioxidant Activity

Antioxidant activity was determined by 1, 1- diphenyl-2-picrylhydrazyl (DPPH), free radical scavenging assay described by Blois [15], with slight modifications. Tea flush extracts (*n* = 3) were diluted with deionized water to obtain concentrations required for DPPH assay. PVPPT standards and tea extracts were subjected to DPPH assay. Test samples (15 *μ*L) were mixed with DPPH reagent (285 *μ*L; 100 *μ*M in absolute ethanol). The mixture was allowed to stand for 30 minutes in dark at room temperature. The control was prepared by adding DPPH reagent (100 *μ*M; 285 *μ*L) to deionized water (15 *μ*L). The absorbance (*A*) was measured using a plate reader at 540 nm and compared with the control. The results were expressed as percentage of inhibition (*I*%) using the following equation:

(1) $$\begin{array}{c} I\%=\frac{\left(A_{control}-A_{sample}\right)}{A_{control}}\times 100. \end{array}$$

### 2.9. Data Analysis

All experiments were carried out at least in triplicate. The effective concentration of the test sample required to scavenge DPPH radical by 50% (EC_50_) was determined by linear regression analysis of the dose response curve obtained between percentage inhibition against concentration. Statistical analysis was performed with Student's *t*-test. Value of *p* < 0.05 was considered as significant. Microsoft Excel was used for all mathematical and statistical calculations.

## 3. Results and Discussion

Polyphenols present in natural products are responsible for the prevention and treatment of diseases [1, 16]. In addition to polyphenols, other categories of active secondary metabolites are terpenes, nitrogen, and sulphur containing compounds [17]. To avoid misinterpretation of results regarding bioactivity of polyphenols, it is vital to develop an effective method to separate polyphenols from other types of secondary metabolites for further analysis for biological activity.

PVPP is an adsorbent use in industry for stabilization of beer, wine, and fruit juices and removal of unsuitable colors [2, 18, 19]. In addition, PVPP has been used for purification of polyphenol oxidase, bioactive compounds from brewery waste streams, and genomic DNA from polyphenol-rich coconut [20–22]. Hou and coinvestigators have employed PVPP suspension with anhydrous sodium sulfate to remove polyphenols from tea to eliminate the matrix effects in determination of insecticides present in tea leaves [23]. Characterization of polyphenols in* Terminalia arjuna* bark extract has been reported after vigorous shaking of the plant extract with PVPP [24]. A recent study demonstrated that the most effective method for polyphenol extraction was PVPP adsorption and liquid–liquid partition with aqueous NaCl, compared to polyamide-SPE extraction and gel permeation chromatography using Sephadex LH-20 [6]. Most methods described the involvement of PVPP suspensions; however the present study needs only a syringe packed with PVPP powder. The whole process can be carried out within 20 minutes.

**Figure 2:**
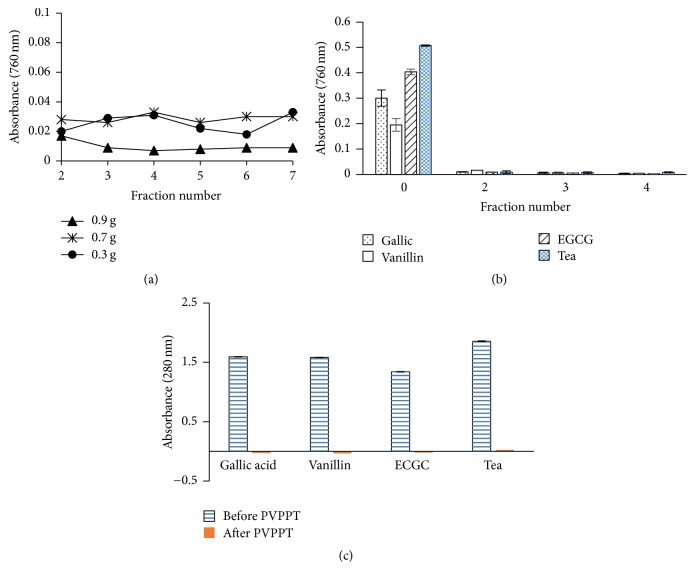
The absorbance (760 nm) obtained for Folin-Ciocalteu assay for polyphenols in different fractions of black tea extract obtained after treatment with PVPP columns containing 0.9, 0.7, and 0.3 g (a), absorbance (760 nm) of gallic acid (100 *μ*g/mL), vanillin (100 *μ*g/mL), epigallocatechin gallate (EGCG) (100 *μ*g/mL), and fresh tea flush (200 *μ*g/mL) after treatment with PVPP column (0.9 g) for Folin-Ciocalteu assay (b), and absorbance at 280 nm for GA (100 *μ*g/mL), vanillin (100 *μ*g/mL), and epigallocatechin gallate (EGCG) (100 *μ*g/mL) (c). Fraction number “0” is the absorbance obtained for fresh tea flush extract before the PVPP treatment. The values are expressed as Mean ± SD. Number of replicates for each experiment are given in the text.

The bud and adjoining two leaves are plucked and processed to manufacture black tea and green tea. Tea leaves* (Camellia sinensis)* contain high amount of polyphenols [25]. In the present study we employed black tea and fresh tea flush to remove polyphenols which have 10.8–12.5 and 20.3–25.6 w/w% gallic acid equivalent, respectively. Dark brown residue appeared on the top part of the column for black tea as shown in the Figure 1. The absorbance found for F-C assay was less than 0.05 at 760 nm after treatment with PVPP at a concentration of 2 mg/mL of black tea (*n* = 6) for all fractions investigated with all quantities of PVPP used (Figure 2(a)). The absorbance remained constant around 0.008 until the 6th fraction obtained from the column packed with 0.9 g of PVPP. An increase in TPC was observed after treatment of black tea extract in the 6th fraction employing 0.3 g of PVPP, reflecting that binding capacity of polyphenols had been exceeded. PVPP column packed with 0.7 g showed similar results as for 0.3 g. Comparing the absorbance values of all fractions of different quantities, the further experiments were carried out with the third fraction collected with 0.9 g of PVPP. Gallic acid, EGCG and vanillin standards, and fresh tea leaves were subjected to polyphenol removal to further validate the reproducibility. Absence of phenolic compounds was also observed with fresh tea flush and standards with the PVPPT samples (Figure 2(b)) in addition to black tea. The values obtained after removal of polyphenols were less than the sensitivity limits of the spectrophotometer for F-C assay which proved the absence of polyphenols. The results were reproducible, independent of tea extracts obtained from different sources or standard phenolic compounds employed. The UV absorbance obtained for PVPPT standards or fresh tea extracts at 280 nm further proved 100% adsorption at a concentration of 100 *μ*g/mL of gallic acid, EGCG, and vanillin as well as for 1 mg/mL of tea flush (Figure 2(c)), suggesting high binding capacities for phenolic compounds. The F-C assay for TPC showed an absorbance of 0.51 at a concentration of 200 *μ*g/mL of fresh tea flush extract compared to an average of 0.008 absorbance for the same of the third fraction with a 0.9 g of PVVP.

**Figure 3:**
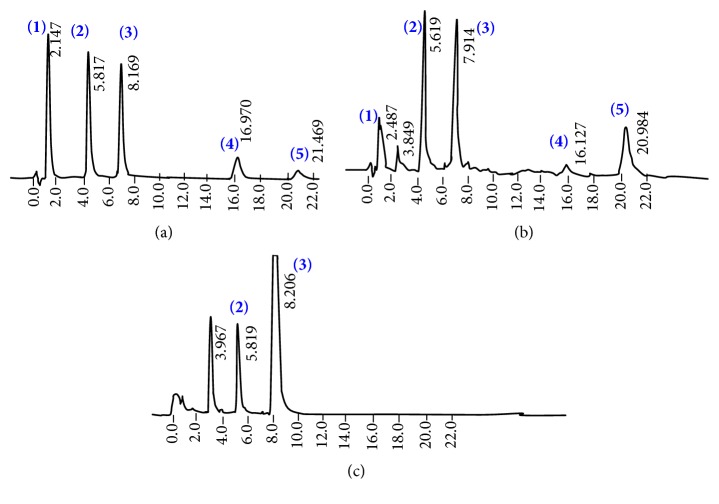
HPLC chromatograms for (a) standard (8 *μ*g/mL), (b) water extracts of tea flush 400 *μ*g/mL, and (c) the third fraction of the tea extract (6.25 mg/mL) treated with PVPP. (1) Gallic acid, (2) internal standard: *β*-hydroxyethyl theophylline, (3) Caffeine, (4) EC, and (5) EGCG.

**Figure 4:**
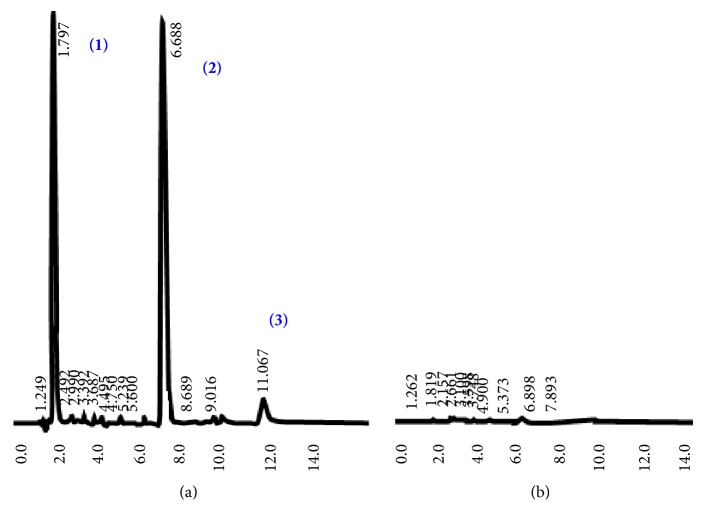
HPLC chromatograms of gallic acid (1), vanillin (2), and epigallocatechin gallate (3) at a concentration of 30 *μ*g/mL before (a) and after (b) PVPP treatment.

Figure 3 shows the HPLC chromatograms of the standard compounds of tea catechins, gallic acid, caffeine (Figure 3(a)), tea extract (Figure 3(b)), and PVPPT tea extract (Figure 3(c)). No peaks appeared in chromatograms relevant to PVPPT catechins, confirming the absence of catechins in the filtrate. However, caffeine, a methyl xanthine, was detected in the filtrate of PVPPT tea extract (Figure 3(c)). Furthermore HPLC chromatograms did not show the presence of phenolic standards in PVPPT samples (Figure 4).

**Figure 5:**
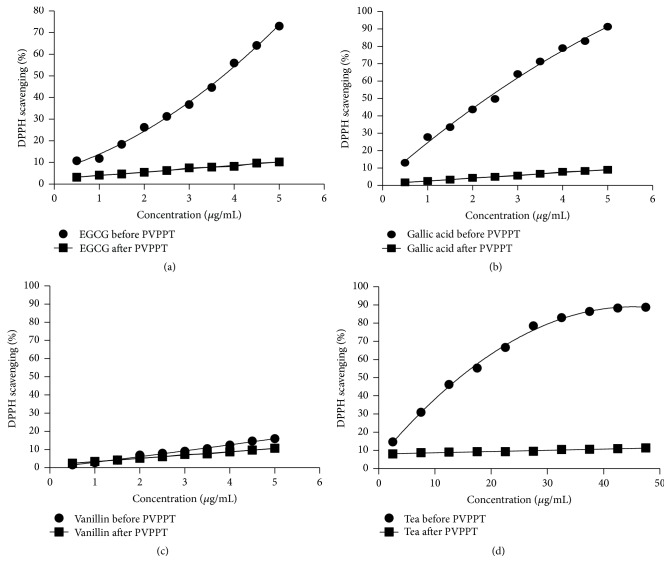
Mean (±SD) percentage of DPPH scavenging capacity of EGCG (epigallocatechin gallate) (a), gallic acid (b), vanillin (c), and fresh tea flush (d), before and after treatment with polyvinylpolypyrrolidone treatment (PVPPT).

The percentage DPPH scavenging capacity of standards used and the fresh tea flush extract before and after PVPP treatment are depicted in Figure 5. The results showed that the percentage of DPPH radical scavenging activity was very high with EGCG, GA, and the tea extract in a concentration dependent manner (Figures 5(a), 5(b), and 5(d)). The percentage DPPH scavenging activity was negligible for the standards and tea extracts with PVPPT samples except for vanillin which did not show an antioxidant activity against DPPH. The EC_50_ values for EGCG and gallic acid were 3.6 and 2.4 *μ*g/mL, respectively. The present study further revealed that vanillin which is a phenolic aldehyde did not show high antioxidant capacity compared to GA and EGCG (Figure 5(c)). The EC_50_ for fresh tea flush extract was 15.5 *μ*g/mL of dry weight.

The technique described above is applied in our laboratory to remove polyphenols from tea products and medicinal plants for wide range of experiments for evaluating biological activity.

## 4. Conclusion

Folin-Ciocalteu assay for total polyphenols, HPLC results, and absorbance values at 280 nm prove that no polyphenolic compounds are present in the PVPP treated filtrate of standard phenolic compounds or tea extracts. The extraction technique described in this paper can be applied to eliminate the polyphenols effectively and efficiently. Furthermore the materials are cheaper and no expensive attachments are used. However it should be noted that the amount of PVPP to be used depends on the content of the polyphenols present in the test samples and a prior experiment should be carried out by changing the quantity of PVPP.

## Abbreviations

PVPP: Polyvinylpolypyrrolidone
EC: Epicatechin
EGCG: (−)-epigallocatechin gallate
DPPH: 1, 1- diphenyl-2-picrylhydrazyl
PVPPT: PVPP treated.
